# Divergent Cardiopulmonary Actions of Heme Oxygenase Enzymatic Products in Chronic Hypoxia

**DOI:** 10.1371/journal.pone.0005978

**Published:** 2009-06-19

**Authors:** Sally H. Vitali, S. Alex Mitsialis, Olin D. Liang, Xiaoli Liu, Angeles Fernandez-Gonzalez, Helen Christou, Xinqi Wu, Francis X. McGowan, Stella Kourembanas

**Affiliations:** 1 Division of Critical Care Medicine, Children's Hospital Boston, Boston, Massachusetts, United States of America; 2 Division of Newborn Medicine, Children's Hospital Boston, Boston, Massachusetts, United States of America; 3 Pulmonary Division, Brigham and Women's Hospital, Boston, Massachusetts, United States of America; 4 Division of Newborn Medicine, Brigham and Women's Hospital, Boston, Massachusetts, United States of America; 5 Division of Cardiac Anesthesia, Children's Hospital Boston, Boston, Massachusetts, United States of America; University of Giessen Lung Center, Germany

## Abstract

**Background:**

Hypoxia and pressure-overload induce heme oxygenase-1 (HO-1) in cardiomyocytes and vascular smooth muscle cells (VSMCs). HO-1^−/−^ mice exposed to chronic hypoxia develop pulmonary arterial hypertension (PAH) with exaggerated right ventricular (RV) injury consisting of dilation, fibrosis, and mural thrombi. Our objective was to indentify the HO-1 product(s) mediating RV protection from hypoxic injury in HO-1^−/−^ mice.

**Methodology/Principal Findings:**

HO-1^−/−^ mice were exposed to seven weeks of hypoxia and treated with inhaled CO or biliverdin injections. CO reduced right ventricular systolic pressure (RVSP) and prevented hypoxic pulmonary arteriolar remodeling in both HO-1^−/−^ and control mice. Biliverdin had no significant effect on arteriolar remodeling or RVSP in either genotype. Despite this, biliverdin prevented RV failure in the hypoxic HO-1^−/−^ mice (0/14 manifested RV wall fibrosis or thrombus), while CO-treated HO-1^−/−^ mice developed RV insults similar to untreated controls. *In vitro*, CO inhibited hypoxic VSMC proliferation and migration but did not prevent cardiomyocyte death from anoxia-reoxygenation (A-R). In contrast, bilirubin limited A-R-induced cardiomyocyte death but did not inhibit VSMC proliferation and migration.

**Conclusions/Significance:**

CO and bilirubin have distinct protective actions in the heart and pulmonary vasculature during chronic hypoxia. Moreover, reducing pulmonary vascular resistance may not prevent RV injury in hypoxia-induced PAH; supporting RV adaptation to hypoxia and preventing RV failure must be a therapeutic goal.

## Introduction

Chronic hypoxia causes remodeling of the pulmonary vasculature with increased proliferation and migration of vascular smooth muscle cells (VSMC), increased pulmonary vascular resistance, increased pulmonary artery pressure, and right ventricular hypertrophy. This clinical condition of “pulmonary hypertension” ultimately leads to death as the right ventricular hypertrophy progresses to dilation and failure. Heme oxygenase-1 (HO-1), an inducible enzyme which degrades the oxidant heme to produce equimolar products of carbon monoxide (CO), biliverdin, and ferrous iron, has been reported to protect animals from proliferation of VSMC [1], [2], apoptosis of cardiomyocytes [3], hypertrophy of cardiomyocytes [4], [5], and cardiac ischemia –reperfusion injury [6]–[8] in a variety of *in vitro* and *in vivo* studies.

HO-1 is known to be potently induced in VSMC under conditions of hypoxia [9], [10] and increased shear stress [11], and in cardiomyocytes under conditions of hypoxia and pressure overload [12]. We have previously reported that HO-1^−/−^ mice exposed to seven weeks of chronic hypoxia develop pulmonary vascular remodeling which is similar to wild-type mice, but their right ventricle (RV) develops a more severe injury pattern characterized by areas of wall fibrosis, apoptosis, lipid peroxidation, and mural thrombi [13]. Although the lack of HO-1 is not associated with worsened pulmonary vascular remodeling in response to hypoxia, constitutive overexpression of HO-1 by type II pneumocytes reduces hypoxic pulmonary vascular remodeling [14]. In the heart, cardiac-specific overexpression of HO-1 protects the myocardium from ischemia-reperfusion injury [6]. Taken together, these data suggest that HO-1 and its enzymatic products provide protection of both the myocardium and the lung vasculature under conditions of hypoxia.

The protective effects of HO-1 are likely the result of the action of its enzymatic products, CO and biliverdin, which is converted by biliverdin reductase to bilirubin. CO activates guanylyl cyclase to produce cGMP, a vasorelaxant second messenger molecule with anti-thrombotic properties [15], [16]. CO also has anti-inflammatory actions and is anti-proliferative in VSMC [17]–[20]. Inhaled CO has been shown to be protective in animal models of inflammatory conditions such as sepsis, hyperoxic lung injury [21], ventilator-induced lung injury [22], and transplant rejection [23]. More recently, Zuckerbraun, et al. have reported that intermittently inhaled CO reverses pulmonary hypertension in different animal models, including hypoxia-induced pulmonary hypertension in mice and rats [24]. In the heart, CO delivered by a CO releasing molecule has been shown to reduce infarct size in mouse and rat models of cardiac ischemia-reperfusion [25], [26].

Increasing evidence suggests that bilirubin and biliverdin may have protective effects that rival those of the better-studied HO-1 product, CO. Biliverdin and bilirubin are potent antioxidants that have anti-inflammatory properties and also reduce vascular intimal growth and wound migration in models of vascular injury [27]–[30]. Injected biliverdin hydrochloride (BV), which is rapidly converted to bilirubin *in vivo* [31], has been shown to be protective in models of cardiac transplant rejection [31], vascular intimal injury [32], and sepsis-induced acute lung injury [33]. In the heart, bilirubin ameliorated postischemic myocardial dysfunction and infarct size in response to ischemia-reperfusion in an isolated-perfused rat heart model [34].

Since HO-1 deficiency results in the characteristic pattern of right heart fibrotic injury with overlying mural thrombus and failure in response to chronic hypoxia, we sought to identify the enzymatic product of HO-1 that is critical for cardioprotection under hypoxia. The main findings of our study are that biliverdin treatment protects the HO-1^−/−^ mouse from RV injury and an exaggerated increase in RV weight after seven weeks of chronic hypoxia without diminishing pulmonary hypertension. In contrast, HO-1^−/−^ mice treated with inhaled CO are protected from pulmonary vascular remodeling, however, they still develop RV failure and thrombus with significant mortality, despite normal right ventricular pressures. The divergent effects of two enzymatic products of HO-1 in the same disease model highlight the complexity of HO-1's protective actions in the cardiovascular system. Moreover, the finding that CO protected from pulmonary hypertension but failed to protect from RV injury indicates that hypoxia has a direct effect on the right ventricle that is not mediated by pulmonary vascular constriction or remodeling.

## Methods

### Animal Model and Hypoxia Exposure

HO-1^−/−^ mice have been previously described [13]. Controls were HO-1^+/−^ littermates as these mice do not develop the RV pathology seen in HO-1^−/−^ mice after 7 weeks of hypoxia (data not shown). All animal experiments were approved by the Children's Hospital Animal Care and Use Committee.

Mice between 8–12 weeks of age were exposed to normobaric hypoxia at 8–10% O_2_ with or without 20–60 ppm CO in a plexiglass chamber where gas delivery is controlled by an OxyCycler (BioSpherix, Redfield, NY). Ventilation is adjusted so that CO_2_ does not exceed 5,000 ppm (0.5%) and ammonia is removed with charcoal filtration. Animals were pretreated with either inhaled CO (20–60 ppm) or biliverdin IX hydrochloride (Frontier Scientific, Logan, UT) (50 µmol/kg ip) one hour prior to the experiment and them maintained in continuous CO or daily BV (or PBS vehicle) injection. CO-oximetry was performed weekly using sentinel animals. Cages were changed and food and water replenished weekly for all animals.

### RVSP Measurements

Mice were anesthetized with pentobarbital (60 mg/kg) and remained spontaneously breathing. A transverse incision was made in the abdominal wall, a 23-gauge needle with tubing attached to a pressure transducer was inserted through the diaphragm into the RV, and pressure was recorded with PowerLab monitoring hardware and software (ADInstruments, Colorado Springs, CO). Animals with heart rates less than 300 BPM were excluded. Mean RVSP over the first ten stable heartbeats was recorded.

### Histological Analysis and RV Weight Measurements

Mice were anesthetized as described above and perfused through the RV with PBS. After inflation of the lungs under constant pressure (15–20 cm H_2_O) with 4% paraformaldehyde (PFA), lungs and hearts were removed and postfixed in PFA overnight. Tissues were paraffin-embedded and 5 µm thick sections obtained for histological analysis. For RV weight measurements, hearts were removed before fixation and both ventricles and LV+septum weighed. Heart weight was normalized for animal weight differences.

For pulmonary histology, H&E stained sections were analyzed and 50–100 µm arterioles were captured with a microscope digital camera system (DXM1200F, Nikon, Japan), and areas obtained using computer-based analysis (NIH Image 1.55). Percentage wall thickness (%) = area_ext_ – area_int_/area_ext_ ×100 where area_ext_ and area_int_ are the area bounded by external and internal elastic lamina, respectively.

For cardiac histology, sections were stained with H&E and Masson's trichrome to determine RV fibrosis or analyzed with terminal deoxynucleotide transferase-mediated dUTP nick end-labeling (TUNEL) to detect DNA breaks in apoptotic cells *in situ*. Hearts were considered positive for RV fibrosis if they contained an area staining positive for Masson's trichrome which extended from the inner aspect to the outer aspect of the RV free wall.

### Cardiomyocyte (CM) Isolation and Anoxia-Reoxygenation (A-R) Exposure

Neonatal ventricular CMs were isolated from 1 day-old Wistar rats and cultured using a commercially available system (Cellutron, Highland Park NJ). Contaminating cardiac fibroblasts were removed by pre-plating cells for 2 hours in uncoated culture flasks. CMs were plated at a density of 25,000 cells/well in 96-well plates. After 48 hours of culture, media were changed in a hypoxic (<1% O_2_) workstation (Ruskinn Technologies, Ltd., Bridgeend, UK) to glucose-free substrate deprivation media pre-equilibrated in hypoxia for 18 hours. CMs were treated with bilirubin HCl (Sigma-Aldrich St. Louis, MO) in PBS or the CO-releasing molecule tricarbonyldichlororuthenium (II) dimer (CORM-1) (Sigma-Aldrich, St. Louis MO) in DMSO or with vehicle control. CO gas at 250 ppm (21% O_2_, 5% CO_2_, balance N_2_) was also used to treat CMs on a separate plate for one hour prior to A-R using Billups chambers (Billups-Rothenberg, Inc., Del Mar, CA). Conditions were replicated in 4–6 wells per plate. Plates were placed in anoxic bags (BD Biosciences, Sparks MD) or left normoxic (controls) and cultured at 37° C for 6 hours. Media were changed to normoxic glucose-containing media with the same concentrations of BR, CORM I, or vehicle and incubated at 37° C for an additional 42 hours.

### Cardiomyocyte Cell Viability/Death Assay

Calcein and ethidium staining was performed using a commercially available viability/cytotoxicity assay (Molecular Probes, Eugene OR). Plates were read on a Packard Fusion fluorescence platereader (Perkin Elmer, Wellesley MA). Calcein to ethidium fluorescence ratio was calculated for each well. Lactate Dehydrogenase (LDH) was also measured using a commercially available kit (Sigma, St.Louis).

### Cardiomyocyte Apoptosis Assay

CMs were exposed to A-R as described above except that reoxygenation was 20 hours. CMs were collected and stained for phosphatidylserine (PS) using FITC-labeled Annexin V. Disruption of CM membranes was detected using 7-alpha actinomycin D (7AAD) (Invitrogen, Carlsbad, CA). Labeling was assessed with a Dako Cytomation MoFlo flow cytometer (Dako, Glostrup, Denmark). Quadrants were defined using unstained and single-stained cell populations. CMs staining positive for PS but negative for 7-AAD were counted and presented as a proportion of all CMs in the sample.

### PASMC Proliferation

Rat PASMC (passage<15) were plated on 96-well plates and serum-deprived for 72 hours. Platelet-derived growth factor (PDGF) stimulated (25 ng/mL)- or unstimulated controls were exposed to 1% O_2_ in a hypoxia workstation in the presence or absence of 1 or 5 µM BR or PBS vehicle. Other cells were exposed to 1% O_2_ and 250 ppm CO inside an airtight Billups chamber. Hypoxic media from the workstation and the Billups chambers were analyzed for pO2 levels using a blood gas analyzer and were found to range between 14 and 25 mmHg in both chambers. After 21 hours, BrdU was added for the final 3 hours of incubation. BrdU incorporation was assessed using a commercially available ELISA (Roche Diagnostics, Mannheim, Germany).

### PASMC Migration

PASMCs were incubated as above in normoxia or 1% hypoxia with or without 250 ppm CO or 5 µM BR for 18 hours before counting and plating in triplicate on the inserts of an 8 µm pore Costar Transwell Plate (Corning, Inc., Corning, NY). PDGF (50 ng/mL) was added to the lower chamber. After 6 hours, an acid phosphatase assay for cell number was performed by incubating the transmigrated cells in substrate solution (10 mM P-nitrophenol phosphate (Sigma), 10 mM sodium acetate, 0.1% Triton X-100, pH 5.8) for 1.5 h at 37°C. After addition of 0.05 ml 1 N NaOH to quench the reaction, OD_410_ was measured using a microplate reader.

### Statistics

Graphs and statistics were performed using the GraphPad Prism 4 software and (GraphPad, San Diego, CA). Significance of differences was assessed non-parametrically using Mann-Whitney U test.

## Results

### Inhaled CO but not biliverdin inhibits pulmonary hypertension

**Figure 1:**
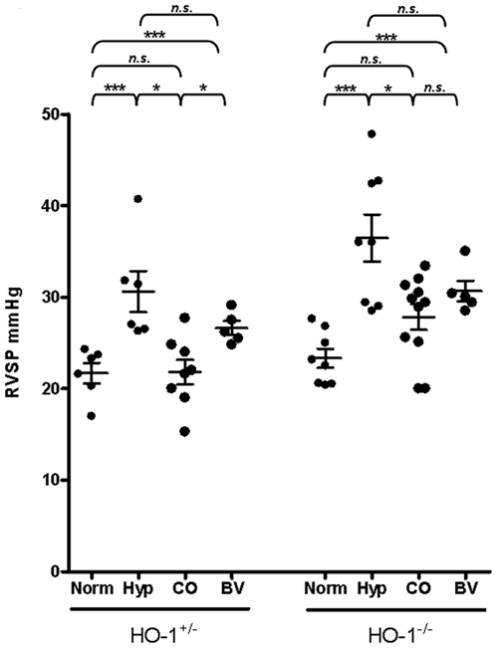
Right ventricular systolic pressure (RVSP) after 7 weeks of hypoxia in HO-1^−/−^ and HO-1^+/−^ control mice. Mean values ^+/−^ SEM are indicated with each dot representing RVSP measurement for one animal. n.s. = not significant, * = p<0.05, *** = p<0.005 as assessed by Mann-Whitney U test.

To assess the impact of inhaled CO and biliverdin injections on the development of pulmonary hypertension, we first measured RVSP in spontaneously breathing mice after 7 weeks of chronic hypoxia. Hypoxia caused a significant increase in RVSP for both HO-1^−/−^ and HO-1^+/−^ control animals, as expected. We were able to use HO-1^+/−^ mice as littermate controls since the HO-1^+/−^ mice manifest the same degree of pulmonary hypertension as wild-type and none develop RV fibrosis or thrombus under hypoxia ([13], and results not shown). Continuously inhaled CO inhibited elevation of RVSP in chronic hypoxia for both HO-1^−/−^ and HO-1^+/−^ animals. For both genotypes, CO treatment resulted in significantly lower RVSP values compared with untreated hypoxic animals and with no significant difference from the values obtained in normoxic control animals. Biliverdin injections did not prevent elevated RVSP for either HO-1^−/−^ or HO-1^+/−^ control animals, as biliverdin-treated animals of both genotypes had RVSP which was significantly higher than normoxic controls but not significantly different from untreated hypoxic mice (Figure 1).

**Figure 2:**
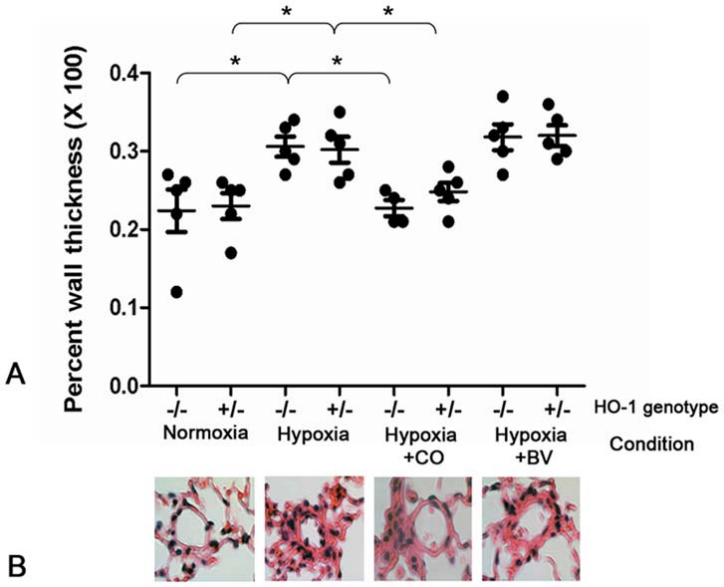
Pulmonary arteriolar remodeling in HO-1^−/−^ animals exposed to chronic hypoxia for seven weeks and treated with CO or Biliverdin, as indicated. (A) Quantification of percent wall thickness. Bars represent means ^+/−^ SEM. Each dot represents one animal and ten vessels were averaged for each animal. * = p<0.05 as assessed by Mann-Whitney U test. (B) Representative pulmonary arterioles of HO-1^−/−^ mice stained with H&E for each of the different conditions and treatments above.

Exposure to chronic hypoxia caused significant pulmonary arteriolar wall remodeling in both the HO-1^−/−^ and HO-1^+/−^ animals. Treatment with continuous inhaled CO for 7 weeks prevented arteriolar wall thickening in both HO-1^−/−^ and HO-1^+/−^ mice, while biliverdin injections had no effect on pulmonary arteriolar remodeling for either genotype (Figure 2). In follow-up experiments HO-1^−/−^ and HO-1^+/−^ animals were treated with intermittent inhaled CO at 250 ppm for one hour per day throughout a seven-week hypoxic exposure. Intermittent CO was equally efficacious as continuous CO in preventing arteriolar remodeling and elevated RVSP during chronic hypoxia (data not shown).

### Biliverdin but not inhaled CO prevents RV fibrotic injury in HO-1^−/−^ mice exposed to chronic hypoxia

**Figure 3:**
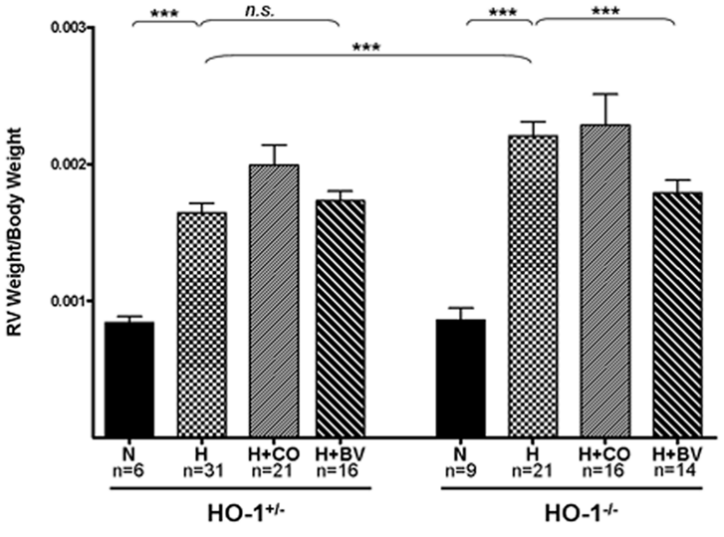
Right ventricular weight in HO-1^−/−^ and HO-1^+/−^ control mice after 7 weeks of hypoxia. RV weight is indexed to animal weight. Means ^+/−^ SEM are shown for the number of animals listed below each column. n.s. = not significant, *** = p<0.005 as assessed by Mann-Whitney U test. N = normoxia, H = hypoxia.

To assess the impact of inhaled CO and injected biliverdin on the development of RV injury in HO-1^−/−^ mice, we weighed hearts and evaluated them grossly and histologically. Hypoxic exposure caused HO-1^−/−^ mice to have a more profound elevation in RV weight (normalized to animal weight) as compared with HO-1^+/−^ control animals (Figure 3). Daily injections of biliverdin (50 µmol/kg) prevented the exaggerated RV weight gain in the HO-1^−/−^ mice, while having no effect on the RV weight of the HO-1^+/−^ control animals. Treatment with continuous inhaled CO throughout the hypoxic exposure did not prevent the elevation in RV weight in the HO-1^−/−^ animals and had no significant effect on the RV weight of the HO-1^+/−^ control animals (Figure 3).

**Figure 4:**
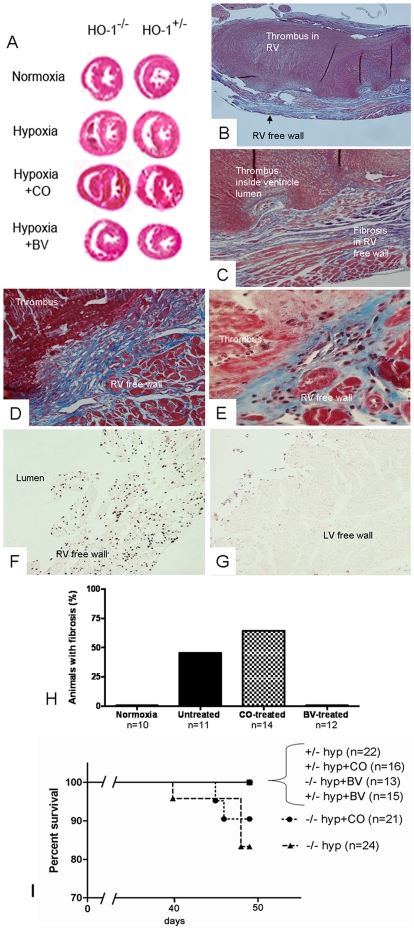
Right ventricular wall injury in HO-1^−/−^ mice exposed to chronic hypoxia for seven weeks. (A) Biventricular (short-axis) sections of the heart at the papillary muscle level stained with H&E reveal RV dilation and thrombus in the hypoxic HO-1^−/−^ controls and the CO-treated hypoxic HO-1^−/−^ mice but not the hypoxic HO-1^−/−^ mice treated with BV. (B,C) Masson's trichrome stained right ventricular sections from an untreated HO-1^−/−^ mouse at 40× (B) and 100× (C) power. A fibrotic area of RV wall is evident with overlying mural thrombus. (D,E) Carbon monoxide-treated HO-1^−/−^ mouse RVs stained with Masson's trichrome at 100× (D) and 200× (E) power showing an area of RV wall fibrosis and overlying mural thrombus. (F,G) TUNEL-stained RV (F) and LV (G) wall from an area of full-thickness wall fibrosis in an untreated HO-1^−/−^ mouse at 200× magnification. The LV has TUNEL stained luminal debris but is otherwise negative for TUNEL-staining. (H) Quantification of the percentage of HO-1^−/−^ animals in each group developing an area of Masson's trichrome-positive fibrosis spanning the full thickness of the RV wall. (I) Survival curve of HO-1 hemizygous and null mice in 8.5–9% oxygen for seven weeks in response to treatment with CO or Biliverdin.

As we have previously reported [13], exposure of HO-1^−/−^ mice to chronic hypoxia for seven weeks caused areas of full-thickness RV wall fibrosis, with the majority of the animals having an overlying mural thrombus (Figure 4A, B, and C). RV injury was evident in many HO-1^−/−^ mice on gross examination, with white thrombus protruding from the RV once atria were removed and often visible through a translucent, dilated RV. 45.4% of untreated HO-1^−/−^ animals developed an area of full-thickness right ventricular wall fibrosis as detected with Masson's Trichrome stain after seven weeks (Figure 4H). Biliverdin injections entirely prevented this response; no HO-1^−/−^ animals treated with biliverdin injections had full-thickness RV wall fibrosis after seven weeks (Figure 4A and H). Unlike biliverdin, continuous CO administration did not prevent RV wall fibrosis in the HO-1^−/−^ animals (Figure 4D, E), with 64.3% of HO-1^−/−^ animals treated with CO developing injury (Figure 4H). These areas of fibrotic injury in the untreated and CO-treated animals had many TUNEL-positive apoptotic cells throughout (Figure 4F), while left ventricular cardiomyocytes on the same slide are negative for TUNEL staining (Figure 4G). No HO-1^+/−^ control animals developed RV wall fibrosis or TUNEL-positive areas. In follow up experiments we have found that HO-1^−/−^ mice treated with intermittent inhaled CO (250 ppm CO for one hour a day) developed RV wall fibrosis and thrombus at a rate similar to HO-1^−/−^ animals treated with continuous CO and hypoxic HO-1^−/−^ controls (data not shown).

In addition to its impact on RV weight, development of RV injury affected death prior to 7 weeks. Approximately 12–18% of HO-1^−/−^ animals died between the 6^th^ and 7^th^ weeks of hypoxia in the untreated or CO-treated group (Figure 4I). Interestingly, there were no deaths prior to seven weeks in the biliverdin-treated HO-1^−/−^ groups, or in the HO-1^+/−^ controls. Necropsy on the untreated and CO-treated HO-1^−/−^ animals dying in the 6^th^ week showed white thrombus protruding from the RV when the atria were removed.

### CO but not bilirubin inhibits hypoxic PASMC proliferation and migration

**Figure 5:**
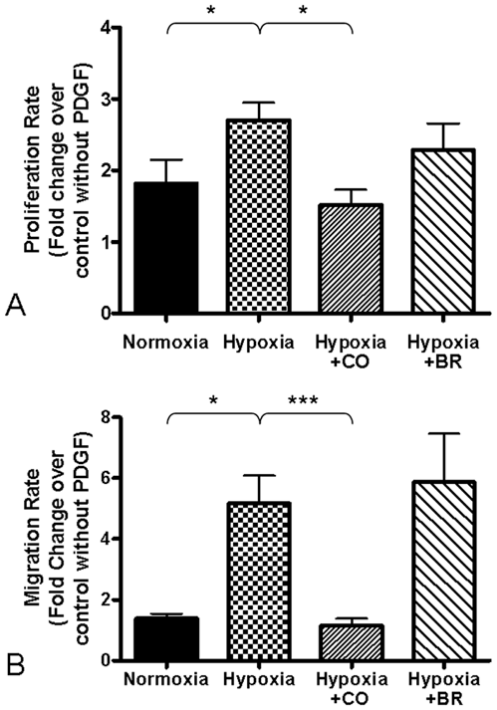
PASMC proliferation and migration in response to hypoxia. (A) Effect of bilirubin and CO on PASMC proliferation in response to hypoxia by BrdU incorporation. Average fold change of relative light units over unstimulated (no PDGF) controls from four experiments are shown. (B) Effect of bilirubin and CO on PASMC migration in hypoxia. Average fold change of relative light units over unstimulated (no PDGF) controls from three experiments is shown. * = p,0.05, *** = p<0.005 as assessed by Mann-Whitney U test.

VSMC exposed to hypoxia have increased proliferation in response to a mitogenic stimulus over normoxic controls, and as we and others have previously reported, treatment of these cells with CO inhibits their proliferation rate [17], [18], [20]. To begin to investigate the differential responses of the pulmonary vasculature to CO and biliverdin, PASMCs were cultured *in vitro* and evaluated for proliferation and migration under hypoxia. Cultured rat PASMCs were treated with either bilirubin (5 µM) or CO (250 ppm) and cell proliferation was assessed in response to PDGF stimulation. The specific doses of bilirubin and CO were selected based on our findings in the cardiomyocyte anoxia-reoxygenation experiments (see below) and our previously published work [17]. We used bilirubin instead of biliverdin in case biliverdin reductase was absent or inactive in the cultured cells. Bilirubin treatment did not affect PASMC growth under normoxia and modestly reduced hypoxia-induced PASMC proliferation (Figure 5A). CO (250 ppm) significantly reduced PASMC proliferation in hypoxia (Figure 5A). PDGF, in addition to being a mitogen, is also a chemoattractant for VSMCs and enhances their migration. Migration of PASMCs through a porous membrane across a PDGF gradient was significantly induced by hypoxia (Figure 5B). Interestingly, CO treatment concurrent with hypoxia completely inhibited hypoxia-induced PASMC migration, whereas bilirubin treatment (5 µM) had no effect on hypoxia-induced migration (Figure 5B).

### Bilirubin protects cardiomyocytes from anoxia-reoxygenation injury

**Figure 6:**
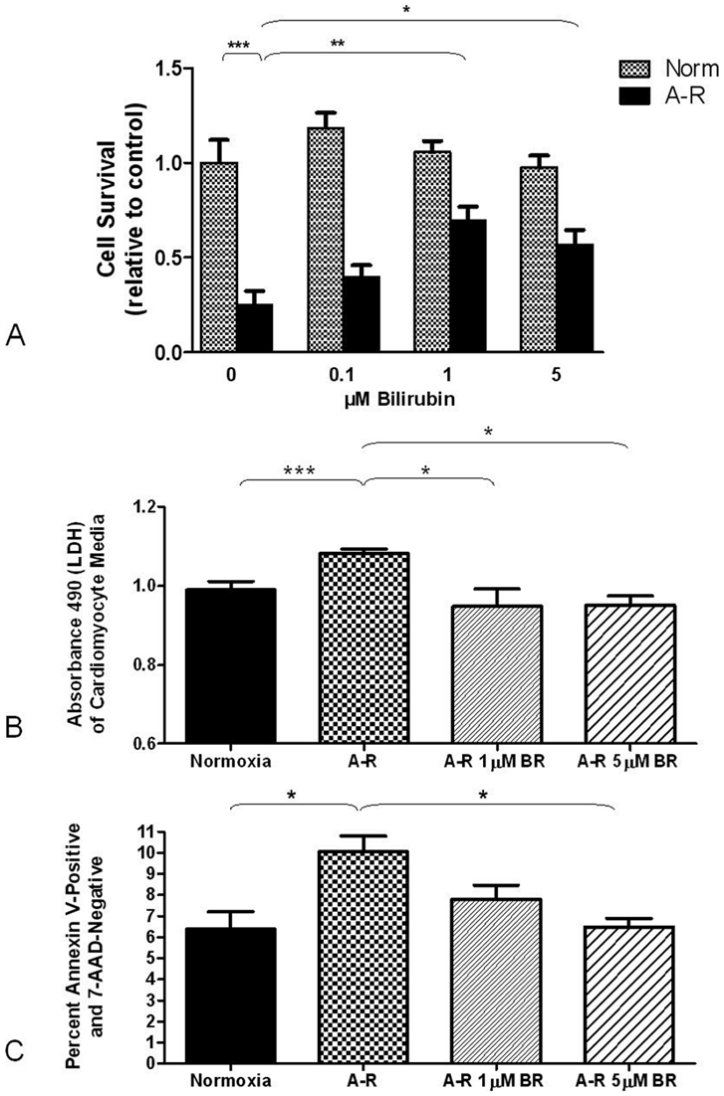
Bilirubin protects cardiomyocytes from anoxia-reoxygenation (A-R) injury and has an anti-apoptotic effect. (A) Cell survival ratio relative to untreated normoxic controls as assessed by calcein/ethidium staining. Mean ^+/−^ SEM for 6 wells per condition is shown and graph is representative of three independent experiments. (B) Lactate dehydrogenase (LDH) measured by absorbance at 490 in the media of cardiomyocytes exposed to A-R. Means ^+/−^ SEM are shown. (C) Flow cytometric analysis of Annexin V-positive, 7AAD-negative cardiomyocytes after A-R. Bars represent the percentage of 10,000 counted cells that fell into this category. Data are an average of three different experiments. * = p<0.05, ** = p<0.01, *** = p<0.005 as assessed by Mann-Whitney U test.

We used an *in vitro* model of A-R to investigate the cytoprotective effects of bilirubin and CO treatment on the cardiomyocyte. Six hours of anoxia followed by reoxygenation results in a significant amount of cell death of cardiomyocytes as assessed by both calcein/ethidium staining (Figure 6A) and LDH (Figure 6B). Neonatal rat cardiomyocytes treated with bilirubin (1–5 µM) demonstrated increased cell survival rate and were better protected against A-R-induced cell death seen in vehicle-treated controls. Higher concentrations of bilirubin (up to 100 µM) were ineffective in protecting the cardiomyocytes, but were not toxic to normoxic control cardiomyocytes (data not shown). Neither CO gas pre-treatment (250 ppm for one hour prior to exposure), nor the CO releasing molecule I (concentrations from 1 to 20 µM) protected cardiomyocytes from cell death (data not shown).

Using flow cytometry for annexin V as an early marker of apoptosis, we evaluated the effect of bilirubin on apoptotic cell death from A-R. To stain dead cells and other cells lacking membrane integrity, 7-AAD was used. Apoptotic cells were identified as those cells staining positive for annexin V and negative for 7-AAD. A-R doubled the percentage of apoptotic cardiomyocytes from the normoxic control levels, and both 1 and 5 µM bilirubin significantly reduced the percentage of apoptotic cells (Figure 6C).

## Discussion

We have used low dose, continuous inhaled CO and injected biliverdin to investigate the role of these enzymatic products in the pathologic RV response resulting from chronic hypoxia and pulmonary hypertension in the HO-1^−/−^ mouse. Inhaled CO prevented pulmonary arteriolar remodeling and reduced RVSP in both HO-1^−/−^ and control animals, while biliverdin had no significant effect on the pulmonary arteriolar response or RV pressure. Although it prevented hypoxia-induced pulmonary hypertension, inhaled CO had no effect on the development of RV fibrotic injury in the HO-1^−/−^ mice exposed to hypoxia for seven weeks. In contrast, biliverdin treatment reversed the exaggerated RV weight response in HO-1^−/−^ mice and completely prevented RV injury at 7 weeks. Given that both molecules have been reported to protect cardiomyocytes and be anti-proliferative for vascular smooth muscle cells in a variety of *in vitro* and *in vivo* injury models, this finding of their differing roles in an *in vivo* model of pulmonary hypertension is intriguing.

Our study is the first to investigate the roles of HO-1 enzymatic products in protection from cardiac injury secondary to chronic hypoxia-induced pulmonary hypertension. Pulmonary hypertension is characterized by pulmonary vascular remodeling that leads to increased pulmonary vascular resistance and elevated pulmonary arterial pressure. Pressure-overload elevates RV wall stress, causing the RV to hypertrophy and ultimately to begin a progression down the end-stage pathway to dilation, fibrosis, apoptosis and failure. Death in patients with pulmonary hypertension is most often a result of right ventricular failure, but mechanisms for progression from RV hypertrophy to failure are poorly understood. The relatively rapid progression to RV dilation, fibrosis, and failure seen in the HO-1^−/−^ exposed to chronic hypoxia makes it an unique tool to understand the roles of HO-1 and its enzymatic products in this injury.

The most striking finding of our experiments is that biliverdin administration completely prevented the characteristic RV injury and failure pattern in the HO-1^−/−^ animals exposed to chronic hypoxia. Biliverdin-treated animals had no areas of full-thickness RV wall fibrosis and correspondingly no significant TUNEL-positive areas of increased RV wall apoptosis. Biliverdin itself is a potent antioxidant because of its ability to be reduced by biliverdin reductase to bilirubin in most tissues. Bilirubin can then be oxidized back to biliverdin, which can re-enter this antioxidant cycle and continue the protection [35]. We have found that injections of 50 µmol/kg of biliverdin in mice increase serum bilirubin levels from a baseline of 0.25 mg/dL to 6.8 mg/dL within 15 minutes of injection, providing evidence for the rapidity of biliverdin reductase activity *in vivo*.

In addition to causing the RV wall fibrotic injury, chronic hypoxia and pressure-overload lead to a heavier, more dilated RV in the HO-1^−/−^ mouse as compared with the hemizygous control animals. This increased weight cannot be explained by a more pronounced hypertrophic response to pulmonary hypertension as the ventricles are dilated, fibrotic, and have mural thrombi. Given the premature death in the untreated and CO-treated HO-1^−/−^ mice, we surmise that cardiac function was significantly impaired as well. Similarly, Hartsfield, et al. reported that although inhibition of HO-1 activity with tin protoprorphyrin did not worsen pulmonary hypertension in rats exposed to hypoxia for 5 weeks, the RV did not hypertrophy appropriately but was dilated and had worsened function as compared with untreated hypoxic controls [36]. In our study, biliverdin treatment prevented the exaggerated RV weight gain seen in untreated HO-1^−/−^ mice, restoring RV weight to HO-1^+/−^ control levels but not preventing the hypertrophic response to hypoxia for either genotype. In sum, biliverdin treatment prevented RV fibrosis and exaggerated weight gain and improved survival in the HO-1^−/−^ mice exposed to chronic hypoxia.

In order to better understand the mechanism behind the myocardial protective actions of biliverdin injection, we used neonatal rat cardiomyocytes to examine the effects of HO-1 products on cell survival after A-R injury. Cardiomyoctes in culture are quite resilient to the effects of chronic hypoxia, but profound hypoxia or anoxia followed by reoxygenation is known to cause cell death. We therefore chose this model to investigate the potential direct effects of CO and bilirubin on cardiomyocytes *in vitro*. We found that cardiomyocytes exposed to A-R had significantly improved survival when treated with low doses of bilirubin (1 and 5 µM). Foresti et al. also found that bilirubin protects cardiomyocytes from A-R *in vitro*, with lower doses of bilirubin (0.5 µM) being protective while higher doses were not [8]. We also investigated whether bilirubin protected from apoptotic cell death and found that 5 µM bilirubin was anti-apoptotic when assessed by flow cytometry for Annexin V. Biliverdin and bilirubin likely protect cardiomyocytes via their antioxidant actions, and given that reactive oxygen species (ROS) are proposed to be mediators of cardiac injury (apoptosis and fibrosis) by pressure-overload, hypoxia, and ischemia-reperfusion, it follows that these molecules could protect the heart simply by their antioxidant properties. Redout and others recently reported that progression from RV hypertrophy to failure in a rat monocrotaline pulmonary hypertension model is associated with increased ROS production by mitochondria and NADPH oxidase [37]. These failing RVs showed evidence of reduced antioxidant capacity and increased oxidative stress. That the antioxidant property of bilirubin is important for cardioprotection is supported by recent data that HO-1 overexpression protected cardiomyocytes from A-R-induced oxidative injury and apoptosis [38]. Our *in vitro* data support an anti-apoptotic role for bilirubin but do not rule out that other protective mechanisms are occurring as well, and these may be occurring via anti-oxidant or other pathways.

Continuous inhaled CO concurrent with exposure to chronic hypoxia completely prevented hypoxia-induced pulmonary vascular remodeling and elevated RVSP. Similar to our finding in mice, others have reported that continuous CO reverses elevations in RVSP in rats exposed to chronic hypoxia for 21 days [39], [40]. We demonstrate here that continuous CO inhalation prevents pulmonary vascular remodeling and elevation of RVSP in mice by chronic hypoxia, and likely does so, at least in part, by prevention of hypoxia-induced pulmonary arterial smooth muscle cell proliferation and migration. Bilirubin, in contrast, only modestly reduced hypoxia-induced PASMC proliferation and had no effect on migration *in vitro*. While we have reported the inhibitory effect of CO on VSMC proliferation in the past, to our knowledge this is the first report of the profound anti-migratory action of CO in hypoxic vascular smooth muscle cells. We have previously reported that the antiproliferative effect of CO in hypoxic VSMC is through cGMP-mediated down-regulation of the cell cycle transcription factor E2F-1 [17], while others have implicated cGMP-dependent phosphorylation of p38 MAP kinase, dephosphorylation of ERK MAP kinase, and regulation of p21 to control cell proliferation [1], [41]. Although the mechanism for hypoxia-induced PASMC migration has not been studied, PASMC migration toward PDGF has been reported to be dependent on ERK MAP kinase phosphorylation [42] and therefore it is plausible that CO inhibits migration via dephosphorylation of ERK 1/2.

A key finding of our study is that despite the protection from pulmonary arteriolar remodeling and elevated RVSP seen in HO-1^−/−^ and HO-1^+/−^ control mice treated with inhaled CO, increased RV weight was not prevented in either genotype and RV fibrosis and apoptosis was not prevented in the HO-1^−/−^ mouse. Other authors who have used CO to treat hypoxia-induced pulmonary hypertension in rats have had conflicting results pertaining to RV hypertrophy. Otterbein, et.al. treated rats with 250 ppm CO once daily during the last three weeks of a six week hypoxic exposure and found improvement in Fulton's Index [24]. Gautier, et.al. treated rats with 50 ppm CO continuously during the last week of a three week hypoxic exposure and found that although RVSP was significantly improved, Fulton's Index was not affected and RV function was significantly worsened in the CO-treated group. In addition, RV infarction developed in some of these animals [39]. For the HO-1^−/−^ mouse that cannot respond to hypoxia by increasing CO production, it appears that neither reducing pulmonary vascular resistance (which occurs as a consequence of CO therapy) nor replacement of CO at the cardiomyocyte level is sufficient to prevent RV injury. Interestingly, for the HO-1^+/−^ control animals treated with CO, elevation of RVSP was prevented but the RV hypertrophied to the same extent as untreated hypoxic HO-1^+/−^ controls. One possible explanation for this effect is CO toxicity, but we monitored COHb levels weekly throughout the hypoxic exposure and found levels less than 9% regardless of CO dose within the range of 20–60 ppm. Others have reported similar COHb levels in rats exposed to chronic hypoxia and continuous inhaled CO at 50 ppm for 3 weeks [39]. These levels are lower than those of chronic smokers and are not thought to be toxic. Another possible explanation is that in hypoxia-induced pulmonary hypertension, CO may be preventing the pulmonary vascular smooth muscle cell proliferative and pro-migratory effects of hypoxia but not protecting from the direct effects of hypoxia on the right ventricle. In support of this, we found that *in vitro*, neither pre-treatment with CO gas nor CORM-1 treatment improved cardiomyocyte cell survival in a model of anoxia-reoxygenation. A previous study by Clark, et.al. showed that CO was protective when delivered using CORM-3 in H9C2 cells exposed to A-R [43]. The use of a different CO-releasing molecule in a transformed cell line (H9C2) in the Clark study may account for the observed difference between their results and ours.

The fact that CO reduced RVSP and pulmonary arteriolar remodeling but did not prevent RV fibrotic injury in the HO-1^−/−^ mice points to a possible direct effect of hypoxia on the RV. When chronic hypoxia is the cause of pulmonary hypertension, the RV is subjected not only to elevated pulmonary vascular resistance from vascular remodeling, but also to systemic hypoxia. Both pressure-overload [12], [44] and chronic hypoxia [13] are known to independently increase HO-1 expression in the heart, pointing to a possible independent role of these two stressful stimuli. As patients with PAH secondary to hypoxia (e.g. secondary to chronic obstructive pulmonary disease) make up a large percentage of patients with PAH, it is important that we investigate this independent effect of systemic hypoxia on myocardial performance and progression to failure.

In summary, biliverdin exerted a direct cardioprotective effect in HO-1^−/−^ mice exposed to chronic hypoxia, but did not ameliorate pulmonary vascular pathology. In the same model, inhalation of low dose continuous CO ameliorated hypoxia-induced pulmonary hypertension in both HO-1^+/−^ and HO-1^−/−^ animals, but did not protect from RV dilation, fibrosis, and mural thrombus. Given that HO-1^−/−^ animals appear to have an accelerated progression to apoptosis, fibrosis, and heart failure, we propose that biliverdin administration slows this progression to resemble the wild-type phenotype while CO either has no effect or accelerates the progression. At the same time, CO dramatically inhibits hypoxia-induced proliferation and migration of vascular smooth muscle cells and prevents hypoxic pulmonary vascular remodeling and RV pressure overload, but has no protective effects on the RV. The distinctly different roles of these two protective enzymatic products of HO-1 in our model of hypoxia-induced pulmonary hypertension are intriguing and may demonstrate how HO-1 can have a variety of protective effects that are dependent on the type of stress and the type of cell involved in injury. As therapeutic strategies that involve upregulation of HO-1 or delivery of its enzymatic products are developed, there will need to be thorough investigation of the possible effects of these strategies in each organ system. In general, our results support the emerging concept that treatments for pulmonary hypertension targeted solely at reducing pulmonary arterial pressure may not necessarily be cardioprotective and may not ultimately improve outcome for these patients. Potential treatments must be evaluated for their protective actions in both organ systems.
